# Mechanism design and human-machine coupling analysis for a lumbar rehabilitation robot

**DOI:** 10.3389/fbioe.2025.1678755

**Published:** 2025-10-01

**Authors:** Yuan Tian, Fengping Chen, Zixu Zhao, Dawei Jiang, Wenjing Ji, Jiasheng Dai

**Affiliations:** ^1^ Electromechanical College, Changchun Polytechnic University, Changchun, China; ^2^ Medical Electrical Testing Laboratory, Jilin Provincial Medical Device Inspection and Research Institute, Changchun, China; ^3^ College of Artificial Intelligence, Changchun University of Finance and Economics, Changchun, China; ^4^ Institute of Technology, Changchun University of Technology, Changchun, China; ^5^ College of Mechanical and Electrical Engineering, Changchun University of Technology, Changchun, China

**Keywords:** lumbar rehabilitation robot, human kinematics, inverse kinematics and statics, superman exercise, lumbar rehabilitation

## Abstract

**Introduction:**

Superman Exercise is highly recommended by healthcare professionals for patients with low back pain. However, performing this treatment can be challenging due to patients’ difficulty in maintaining the training maneuvers and the lack of standardization in these maneuvers.

**Methods and procedures:**

A low back rehabilitation robot, designed based on swallow movement rehabilitation training, was fully modeled for patients with low back motor dysfunction. The robot underwent kinematic analysis, including forward and inverse solutions, as well as kinetic analysis to verify its accuracy. By studying human waist mobility and muscle force, a human-machine coupling model was developed to determine the range of human waist joint angles and muscle force size. This was achieved by importing three-dimensional motion capture data into the OpenSim human motion analysis environment and comparing it with the human body’s natural lumbar rehabilitation movements. Additionally, comparisons were made with the human body during lumbar rehabilitation training maneuvers.

**Conclusion:**

The rehabilitation robot is well-designed to meet patients’ rehabilitation training needs and uncover the rehabilitation patterns of the human waist. This study serves as a reference for future parameter optimization and control system design.

## 1 Introduction

Lower back pain (LBP) is a significant global health issue, characterized by pain, muscle tension, and stiffness from the ribbed edges of the back to the buttock crease. Lumbar Stabilization Exercise (LSE), also known as core stabilization exercise (Hoy et al., 2012), is commonly used in the conservative treatment of LBP. Swallow Lumbar Stabilization Exercise, a key component of early rehabilitation, is particularly beneficial for hemiplegic patients (Moon et al., 2013). This specific type of exercise can aid in remodeling the patient’s nervous system, enhancing balance and strength control, improving muscle control in the core region, and facilitating the restoration of basic physiological lumbar function Hestbaek et al. (2003).

However, challenges exist with lumbar stabilization training, such as maintaining consistency in movements and the need for significant medical rehabilitation resources, and the involvement of rehabilitation professionals, leading to limited rehabilitation effectiveness. To address these issues, the development and implementation of rehabilitation robots are seen as a promising solution to enhance the availability of rehabilitation resources and improve the quality of life for However, challenges exist with lumbar stabilization training, such as maintaining consistency in movements and the need for significant medical rehabilitation resources, and the involvement of rehabilitation professionals, leading to limited rehabilitation effectiveness. Individuals with lower back pain.

Recent years have seen extensive research by experts worldwide on lumbar rehabilitation robots Kim et al. (2022). In 2022, JOOWAN KIM et al. introduced a lumbar spine stabilization training robot, aiming to exercise the lumbar muscles by providing quantitative auxiliary force to the human body. However, challenges arose in maintaining balance during experiments due to ergonomic design issues and clinical requirements not being fully met. In 2019, Zhengmeng Yang et al. designed a lower lumbar cord-driven parallel rehabilitation robot Yang et al. (2019), which guided the human body’s end through a rope drive but lacked the ability to effectively train the lumbar muscles for balance training.

In 2023, Li et al. (2023). Proposed adaptive predictive control to directly support the auxiliary effectiveness and traceability in the task of “load/attitude diversity.” In 2023, Moya-Esteban et al. (2023) esteban and others built a real-time EMG driven muscle bone model to elaborate the quantitative mapping of the relationship between lumbar muscle loads. In 2024, Kim et al. (2024) and others achieved fine-grained and hierarchical adjustment of lumbar muscle load through a series of elastic actuators against gravity. However, some experiments only give the mapping of the relationship between lumbar muscles, and do not participate in the robot design and experiment, which can not achieve a better effect of lumbar muscle training.

Based on the law of lumbar motion, this paper proposes a set of lumbar rehabilitation robot for “Superman Exercise” core stability training. Compared with the common pull type and end guide type devices, this system adopts the “chest + leg” double support electric push rod load sharing configuration, which can reduce the local load of lumbar spine and support the balance training of stabilizing muscle without changing the training intention; The prescription parameterization is introduced into the function to realize the smooth switching of passive auxiliary/active auxiliary/resistance within the unified impedance/force control framework; In terms of availability and safety, the four-tier mechanism of mechanical limit, dual channel emergency stop, soft limit and process grouping is integrated to achieve rapid individualized adjustment. We analyzed the working range and typical rehabilitation state of the system, and quantitatively evaluated the differences of rectus abdominis, multifidus, internal oblique and quadratus lumboris muscles under the condition of “autonomous vs. robot assisted” based on opensim man-machine coupling model; The results show that the system can reduce muscle strength demand and improve endurance while maintaining the mobility of training mode, which provides the basis for subsequent research and clinical prescription.

## 2 Superman exercise robotic system

### 2.1 Structural design

#### 2.1.1 Analysis of superman exercise lumbar rehabilitation training

**FIGURE 1:**
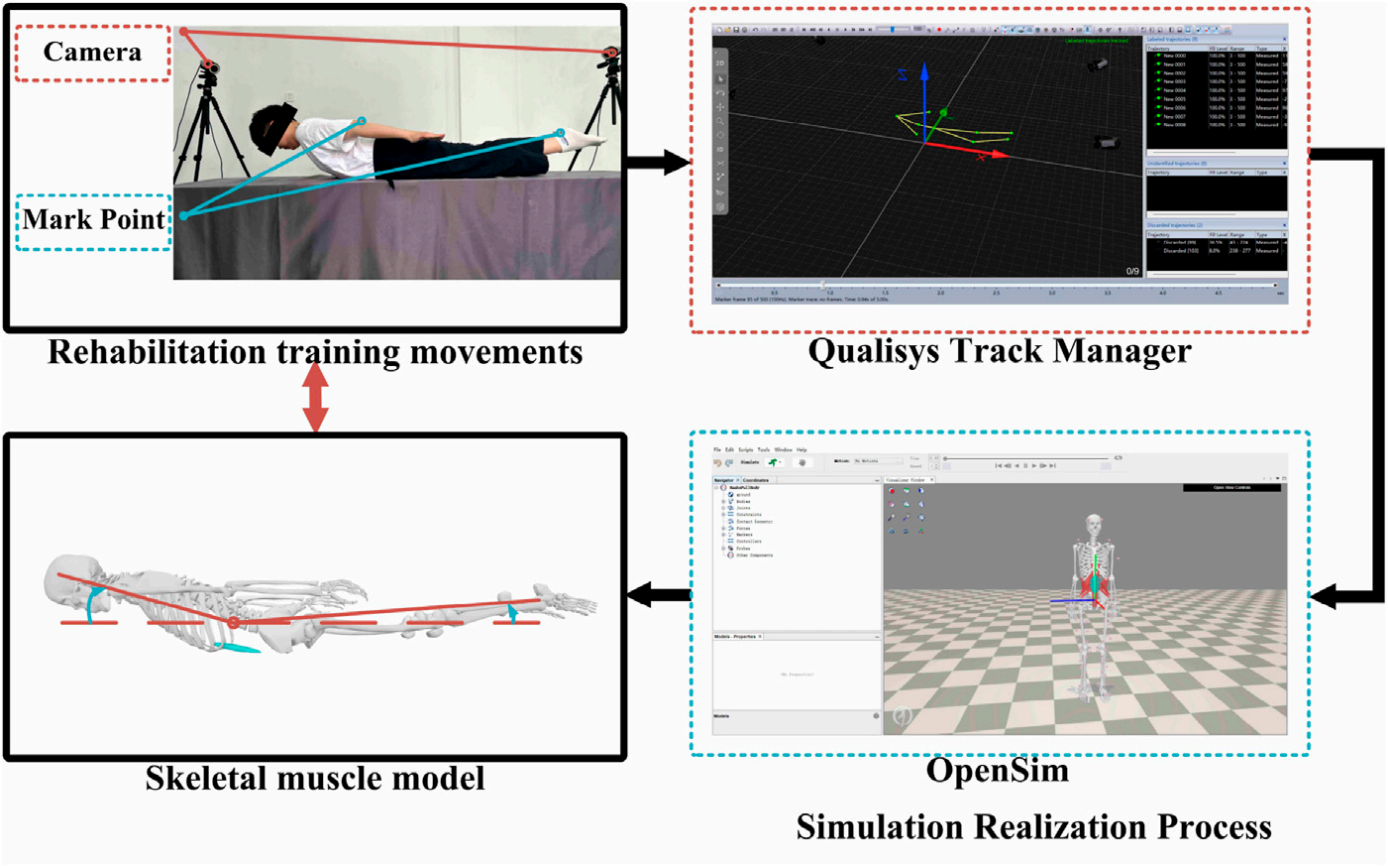
Analysis of swallow rehabilitation training.

Superman Exercise, depicted in Figure 1, is a prevalent method for training lumbar muscles. This exercise targets key muscles like the rectus abdominis, multifidus, Internal Oblique, and Quadratus lumborum, enhancing maximal isometric strength in the lumbar extensors and decreasing pain levels Moya-Esteban et al. (2023). Unlike traditional strength training, Superman Exercise focuses on enhancing muscle endurance and motor balance control. To achieve the desired outcomes, this training should be conducted over a specific timeframe with minimal muscle contraction. However, adjusting limb load to a suitable level can be challenging, particularly for certain patient groups such as the elderly, frail, obese individuals, and those with upper or lower extremity disabilities. These patients may struggle with swallow rehabilitation training, leading to potential muscle overload and injury. To address these challenges, this study employed the Qualisys motion capture system to capture rehabilitation postures, which were then analyzed in OpenSim to assess muscle and bone activity during Superman Exercise. The analysis revealed that the rectus abdominis, multifidus, internal obliques, and Quadratus lumborum play a crucial role in driving the upper trunk and lower legs, facilitating lumbar muscle endurance and balance control during swallow rehabilitation training. To mitigate the risk of lumbar muscle injuries from upper trunk and lower leg weight loads, a specialized swallow-style lumbar rehabilitation training device was developed. This device reduces the strain on lumbar muscles by providing additional support to the upper trunk and lower legs, enabling patients to effectively complete swallow-style lumbar rehabilitation training for improved muscular endurance and motor balance control.Through the chest and leg support structure to reduce the load of lumbar muscle group, effectively avoid muscle overload in the training of weak or elderly patients, and ensure the safety of the training process.

#### 2.1.2 Superman exercise rehabilitation robot mechanical structure design

**FIGURE 2:**
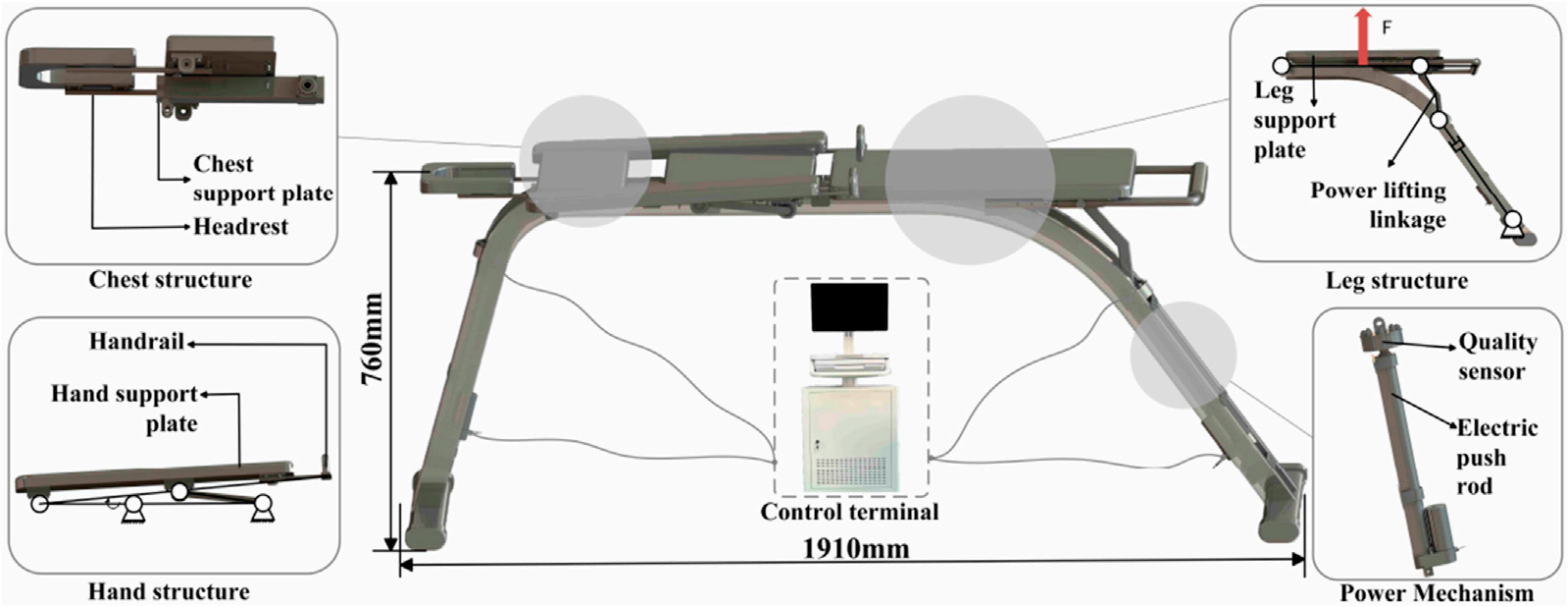
Low back rehabilitation robot.

The Superman Exercise exercise involves bending the lumbar spine, training the upper trunk and lower legs to lift off the ground using lumbar muscle traction. A lumbar rehabilitation robot assists in this exercise by utilizing an electric linear actuators for spatial movement, enabling patients to perform the training. The 3D model of this robot is depicted in Figure 2. Comprising three parts - the main frame, power mechanism, and action support member - the lumbar rehabilitation robot is constructed from welded square tube steel, forming a stable arched base frame. The power mechanism features two sets of electric actuators and powerlifting connecting rods, while the action support member includes structures for the head, chest, abdomen, hands, and legs. The chest and leg structures are driven by an electric push rod, which can assist the movement of the upper trunk and lower limbs while reducing the gravity pressure on the waist, and meet the rehabilitation needs of patients with different constitutions by adjusting the support force. So as to avoid overload and obtain better training effect. The chest and leg electric push rod drive structure can effectively reduce the lumbar load while assisting the trunk and lower limb movement, and achieve individualized adaptation by adjusting the support force, which can not only avoid excessive load and ensure the safety of training, but also have the potential to expand to other spinal rehabilitation scenarios. In order to improve the clinical usability, this system has designed a security mechanism in the four layers of hardware control software process. Hardware layer: the electric push rod and connecting rod are set with mechanical limit, and the contact interface adopts soft gasket and adjustable restraining strap; The whole machine is equipped with dual channel normally closed emergency stop (platform red e-stop and handheld “dead switch”), which will directly cut off the power circuit and enter the safety position in case of power failure or emergency stop. Control layer: the impedance/force control integrated framework is used to set the soft limit and saturation for the auxiliary/resistance (normalized according to the body mass n/kg), joint range of motion (In order to meet the comprehensive needs of low-speed, strong, repeatable prescription and clinical maintenance, the chest/leg support unit uses an electric linear actuator. Compared with the wire/cable drive and pneumatic scheme, the electric push rod is more suitable for the prescription implementation of impedance/force control in the “Superman Exercise” training scenario in terms of controllable position and speed, noise and maintenance costs, power failure self-locking and the integration of software and hardware multi-layer security strategies. The main structure adopts square steel pipe welded frame to obtain sufficient base stability and maintainability; Considering the engineering trade-off between specific stiffness and connection reliability, we use aluminum profile/thin-walled circular tube to reduce the weight of the upper non bearing parts, thus forming a configuration of “stable base + light upper part + compliant contact”.

Figure 1 shows the posture and target muscle group of Superman Exercise lumbar stability training, indicating the main stabilizing roles of rectus abdominis muscle, multifidus muscle, internal oblique muscle and quadratus lumboris muscle in the movement, as the physiological basis for the selection of robot training scenes and indicators.

As shown in Figure 2. Arch bridge shaped square steel base, two sets of electric linear push rods and chest/leg support components; The support force can be adjusted to reduce the lumbar spine load and achieve individualized adaptation without changing the training intention; Supporting mechanical limit and dual channel emergency stop.

#### 2.1.3 Training protocol and parameterization

In order to adapt to different rehabilitation stages and prescription goals, this paper parameterizes the training protocol as four tuples: $\Pi =\left\{Fs,\;ROM,\;\omega ,\;T_{\text{hold}}\right\}$ Of which, Fs Is the equivalent auxiliary/anti resistance (normalized in N/kg) applied by the chest/leg support member to the patient, ROM is the percentage of active backward extension activity of relative individual L1 – S1, ω is the target angular velocity (°/s), and $T_{\text{hold}}$ is the end pose retention time (s). The stroke and force control of electric push rod is used to realize Fs Continuously adjustable; The chest/leg kinematic trajectory and inverse solution target provide rom and ω settings; The control layer adopts impedance (virtual spring damping) to realize three modes of “active auxiliary/passive/resistance”, and sets upper limit and saturation treatment for angle, force and speed to ensure safety.

### 2.2 Kinematic analysis

In this section, computational simulations for the training process of a lumbar rehabilitation robot are conducted. To analyze the robot’s state changes during motion, an efficient mathematical method is required to describe kinematic aspects like displacement, velocity, and acceleration of a rigid body. This study utilizes the matrix method to address the robot’s kinematic challenges. By establishing the reference coordinate system and kinematic coordinate systems for each joint based on the action support members’ positions, we develop the forward kinematics model using the improved DH parametric method. Subsequently, inverse kinematics calculations are performed using the algebraic method Chakraborty and Ghosal (2004), culminating in the deduction of the motion space of the lumbar rehabilitation robot to set the groundwork for subsequent experiments.

#### 2.2.1 Forward kinematics analysis of lumbar rehabilitation

The lumbar rehabilitation robot discussed in this study features a chain structure comprising various joints connected in series, incorporating motion support components like the head, chest, abdomen, hands, and legs. Initially, a mathematical model is developed for kinematic analysis.

**FIGURE 3:**
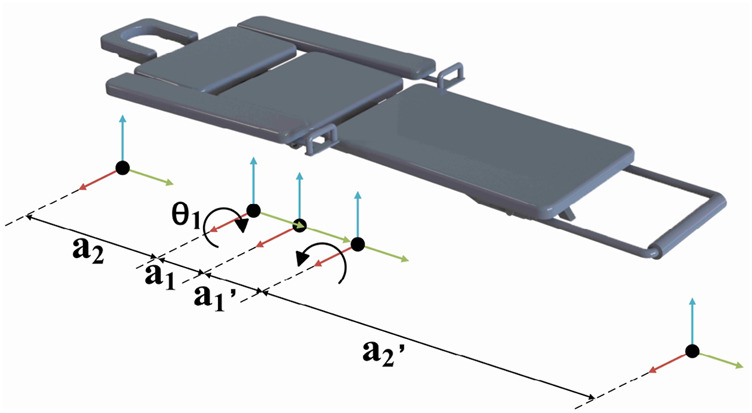
D–H rod model.

The positional analysis of the lumbar rehabilitation robot is conducted based on its position matrix. The position matrix of the connecting rods relies on structural parameters, kinematic configurations, motion parameters, and geometric modeling in various sequences Liu et al. (2005). Beginning with an examination of the robot’s structure, amodified D-H parametric method model is constructed, depicted in Figure 3. The black spot is the origin of the joint; Blue/red/green arrows are z/x/y-axis respectively; The arc arrow indicates the direction of rotational degrees of freedom; $a_{1}$ and $a_{2}$ are the distance between adjacent joints (unit: mm, see Table 1 “connecting rod parameters”). The model is used to derive the forward kinematics and terminal pose transformation matrix.

**TABLE 1:** Parameter Table of the lumbar rehabilitation Robot’s linkage.

Parameter number	α	a	d	θ
1	0	a_1_	0	0
2	0	a_2_	0	θ _1_

The enhanced D-H parameters for each linkage of the lumbar rehabilitation robot, along with the range of variation of joint variables, are determined and presented in Table 1 based on the robot’s structure and kinematic parameters among the primary components.

Substituting the lumbar rehabilitation robot linkage parameters from Table 1 into the improved D-H parameter method allows for the calculation of the transformation matrix of each linkage ${\;\;}_{i}^{i-1}\;T$ (Formula 1) and the total variation matrix of the leg or head end coordinate system concerning the base coordinate system ${\;\;}_{0}^{2}\;T$ , (Formula 2) as shown below.


$${\;\;}_{0}^{1}\;T=\left[\begin{array}{cccc} 1 & 0 & 0 & a_{1}\\ 0 & 1 & 0 & 0\\ 0 & 0 & 1 & 0\\ 0 & 0 & 0 & 1 \end{array}\right]$$ (1)

$${\;\;}_{2}^{1}\;T=\left[\begin{array}{cccc} \cos\theta_{1} & -\sin\theta_{1} & 0 & a_{2}\\ \sin\theta_{1} & \cos\theta_{1} & 0 & 0\\ 0 & 0 & 1 & 0\\ 0 & 0 & 0 & 1 \end{array}\right]$$ (2)

For is obtained by the product of five linkage transformation matrices (Formula 3):

$${\;\;}_{2}^{0}\;T{=}_{1}^{0}\;T\times_{2}^{1}\;T=\left[\begin{array}{cccc} \cos\theta_{1} & -\sin\theta_{1} & 0 & a_{1}+a_{2}\\ \sin\theta_{1} & \cos\theta_{1} & 0 & 0\\ 0 & 0 & 1 & 0\\ 0 & 0 & 0 & 1 \end{array}\right]$$ (3)

**FIGURE 4:**
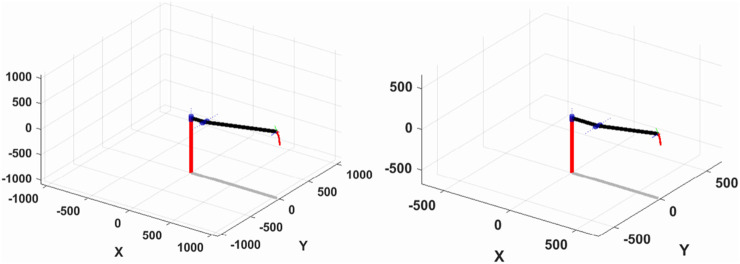
Spatial motion curves of the leg (left) and chest (right) structures.

In this paper, we utilize the Robotics Toolbox in Matlab for simulation and analysis. We program and import each linkage’s coordinate system and its transformations using the improved D-H parametric method into Matlab. brivebot, we generate 3D spatial maps of the mechanical joint motion. Within the joint-type coordinate system, the initial point is defined as q0 = [0,0,0], and the termination point as q1 = [0, pi/6, 0]. The end-pose matrices for both the initial and termination points are calculated using the fkine (r, q) function. Subsequently, the jtraj (r,q) function is used to determine the joint trajectory q = jtraj (q0, q1, t), where t represents the time set for the planned points. Finally, T = fkine (r, q) is called to establish a one-to-one correspondence between the specified points and the 4×4 matrix T. The resulting spatial motion profile, based on the specified initial and endpoints, is illustrated in Figure 4. As shown in Figure 4, the time grid t is uniformly sampled; Blue = leg module, red = chest module; Coordinate unit: mm/°.

**FIGURE 5:**
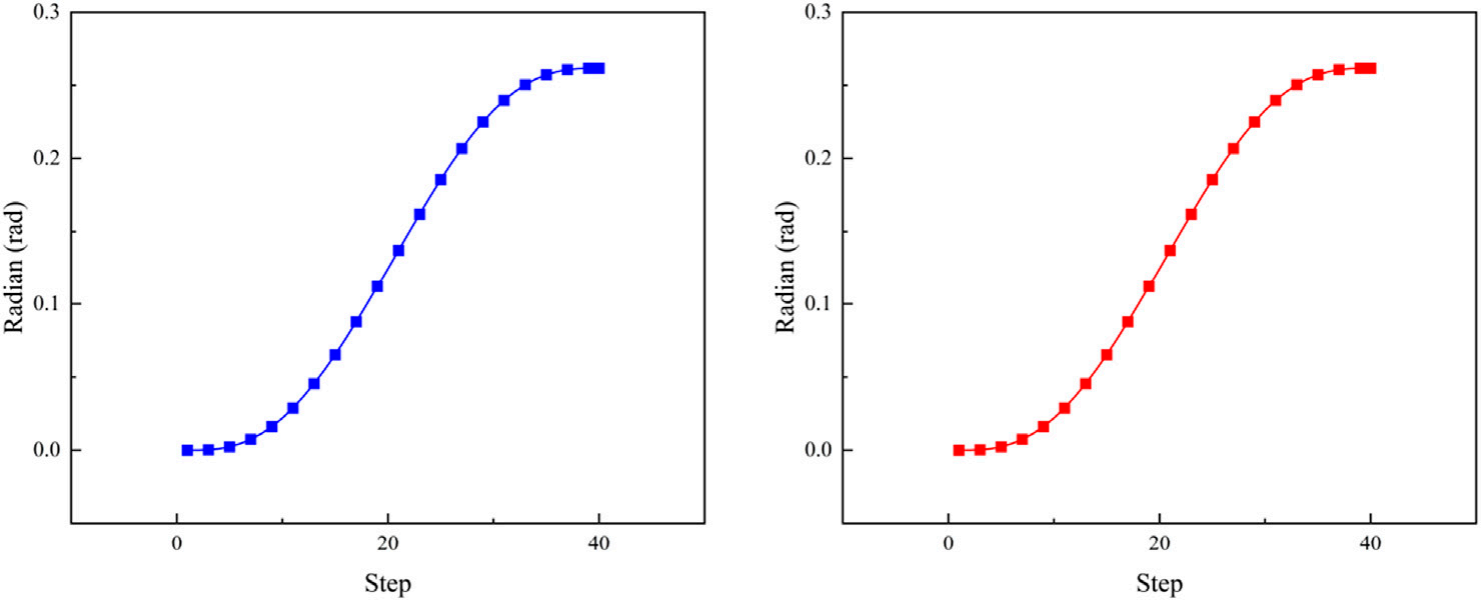
Leg (blue) and chest (red) joint curvature curves.

Figure 5 displays the variation curves of the movable joint angles for the chest and leg structures. It is evident from the figure that the motion curves of each joint angle along the spatial planning trajectory, determined by the initial and termination points, exhibit smoothness and align with the specified range of motion requirements.

#### 2.2.2 Reverse kinematics analysis of lumbar rehabilitation

The kinematics problem is crucial in robot coordinates, particularly the inverse differential kinematics problem for a tandem chain operator. This involves determining the joint motion rates based on unit positions in the link and combined end velocities. Three primary methods exist for solving robot inverse kinematics: geometric, analytical, and numerical iterative methods Paul (1981), Paul and Shimano (1979). The geometric and analytical methods may lack constraints for a unique joint solution, whereas the numerical iteration method decomposes end trajectories into microelements to iteratively derive a unique value Li et al. (2022).

The lumbar rehabilitation structure in this study prioritizes the overall motion angle of the patient’s lumbar spine rather than the precise spatial localization of mechanical structure endpoints. It focuses on the angle formed by joint tangent lines at both ends of the patient’s lumbar spine in the coordinate plane, emphasizing consistency in positive and negative kinematic solutions during inverse motion simulation Karlik and Aydin (2000).

**FIGURE 6:**
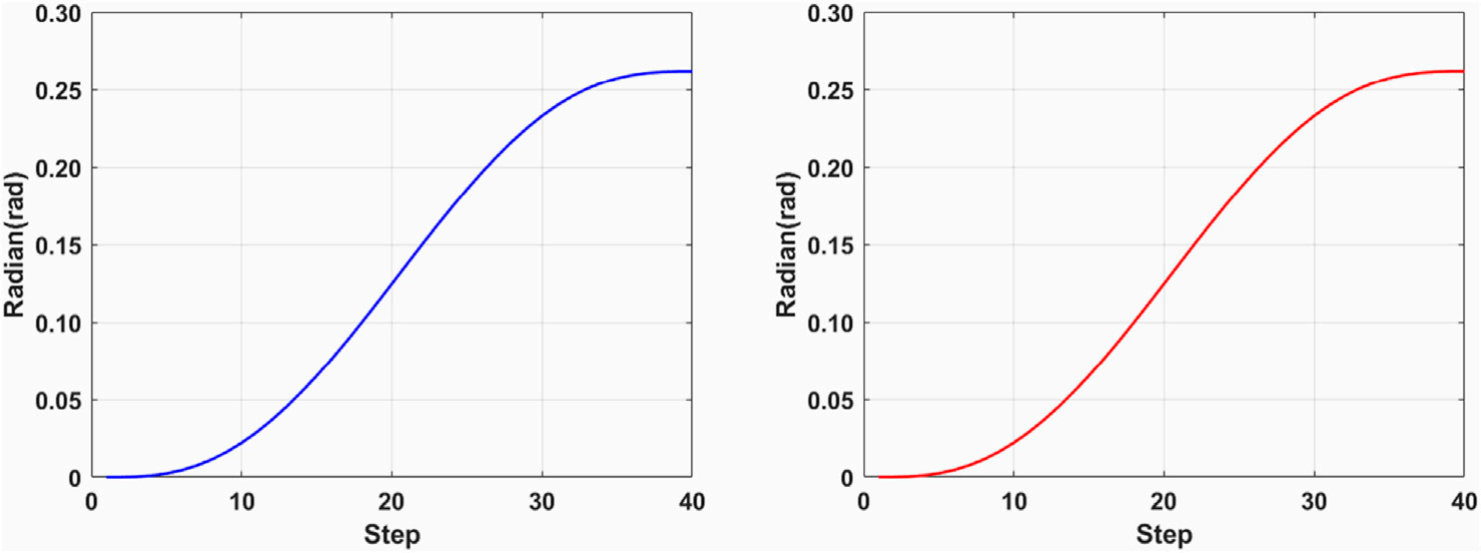
Inverse kinematics leg (blue) and chest (red) joint curvature curves.

Building upon forward kinematics simulation, the ikine function in the Robotics Toolbox is employed to conduct inverse kinematics simulation using the same initial and termination points. A comparison with the forward kinematics solution reveals consistent curves in both forward and inverse kinematic simulations, as depicted in Figure 6.

### 2.3 Dynamic analysis

This chapter focuses on analyzing the mechanical properties of the mechanism, specifically addressing force analysis. Initially, clearance within the moving pair is omitted, with the discussion centered on force analysis of a planar mechanism comprising rigid members. The D’Alembert’s principle is commonly applied for this purpose, treating inertia force and moment of inertia as external forces and moments. Consequently, the moving mechanism is considered in a state of static equilibrium, enabling analysis through statics methods Davidson et al. (2004). This approach is known as dynamic static analysis of the mechanism.

#### 2.3.1 Upper limb dynamics analysis of lumbar rehabilitation robot

**FIGURE 7:**
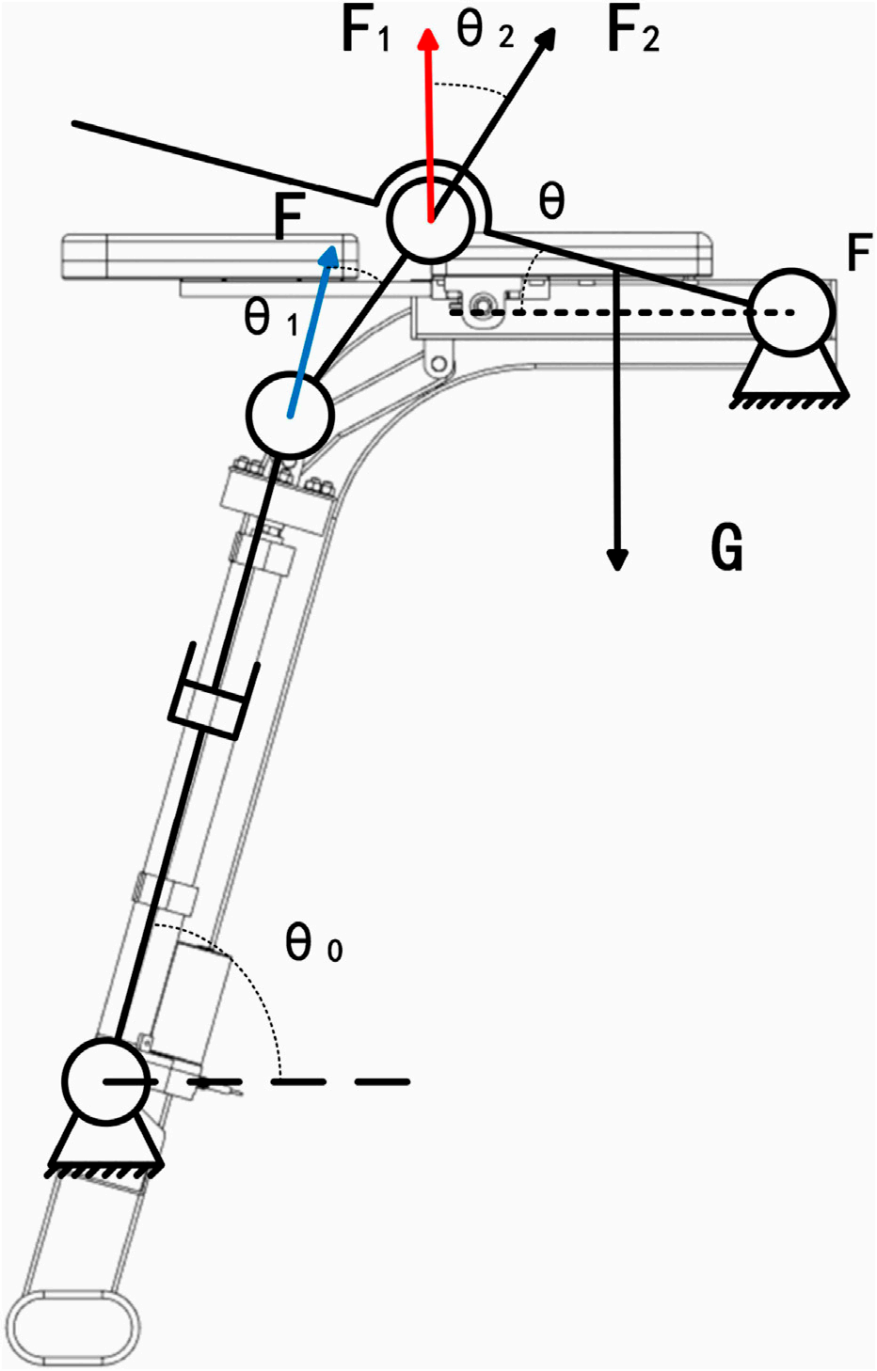
Mechanical sketch of the upper limb of the lumbar rehabilitation robot.

The diagram in Figure 7 illustrates the mechanical structure of the upper limb in the lumbar rehabilitation robot.

The lumbar rehabilitation robot operates primarily through the axial movement of the electric actuator mounted on the frame. This movement propels the powerlifting linkage, transferring power to the upper limb movement plate to facilitate human body motion. Where θ is the attitude angle of the chest motion board relative to the horizontal. G is the equivalent gravity of the chest movement board and the upper chest cushion (N); F is the axial force of electric linear push rod (n), blue arrow; $F_{1}$ , $F_{2}$ , are the normal/tangential component force (n) acting on the hinge point of the chest support plate, with red/black arrows. $\theta_{0}$ , is the installation angle between the push rod and the base; $\theta_{1}$ , is the angle between the push rod and the connecting rod; $\theta_{2}$ , is the relative horizontal posture angle of the chest motion board.

The support plate is obtained at rotational speed ω , after the motion time (Formula 4);

θ=ω⋅t (4)

First, calculate the change in the angle of each part of the lifting mechanism due to the upper limb support plate (Formulas 5–7):

$$\cos\left(2/\pi -\theta_{0}\right)=\frac{AB}{AD}$$ (5)

$$\tan\left(2/\pi -\theta_{0}\right)=\frac{BD}{AB}$$ (6)

$$\cos\theta =\frac{DE^{2}+CE^{2}-CD^{2}}{2DE\cdot CE}$$ (7)

Where A is the electric actuator endpoint, B is the horizontal intersection of the electric actuator endpoint vertically and the fixed end of the support plate, C is the connection point of the connecting rod and the support plate, D is the horizontal intersection of the connecting rod and the fixed end of the support plate, and E is the fixed end of the support plate.

As the leg plate ascends gradually and steadily, the consideration of inertia force and moment of inertia is disregarded. This allows for the determination of the axial force point of the push rod to establish the equilibrium program (Formula 8).

$$G\cdot a_{1}=F_{1}\cdot a_{2}$$ (8)

where $a_{1}\;a_{2}$ is the horizontal distance from the center of mass of the leg plate to E and the horizontal distance from point D to point E, respectively (Formulas 9–11);

$$F^{\prime }=F\cdot \cos\theta$$ (9)

$${\;F}_{1}=F^{\prime }\cdot \cos\theta_{2}$$ (10)

$$\theta_{2}=\left(2/\pi -\theta_{0}-\theta_{1}\right)$$ (11)

where $F^{\prime }$ is the component of F on the power lift linkage, and in summary, the axial force F on the upper limb electric actuator can be derived as Formula 12;

$$F=\frac{G\cdot a_{1}}{\cos\theta \cdot \cos\theta_{2}\cdot a_{2}}$$ (12)

#### 2.3.2 Lower limb dynamics analysis of lumbar rehabilitation robot

**FIGURE 8:**
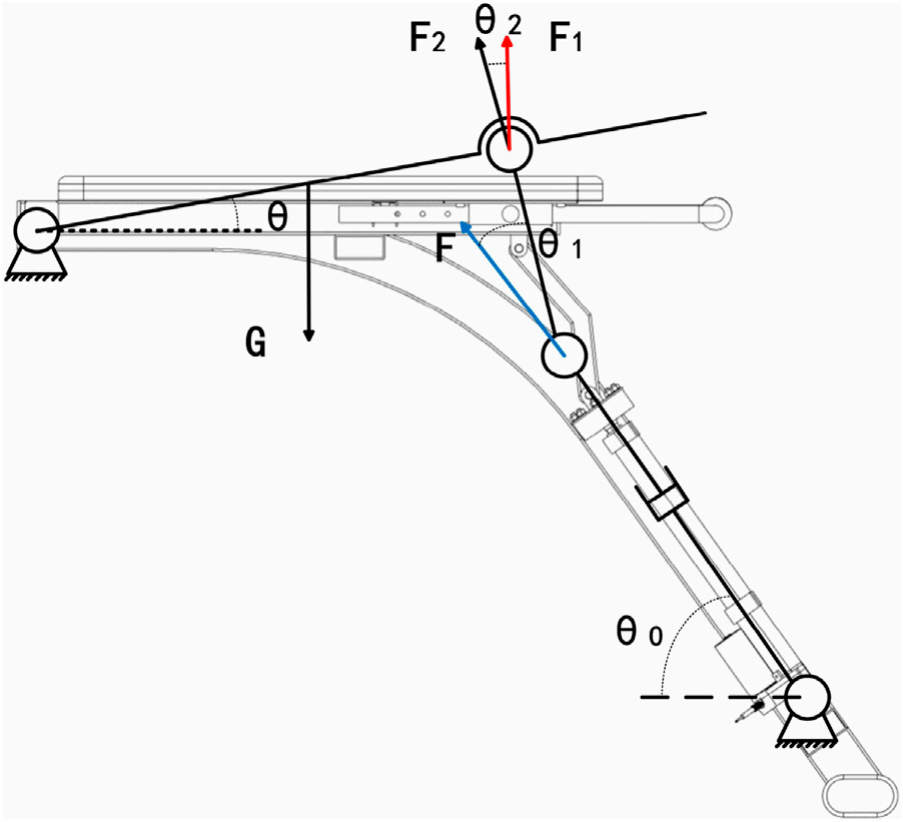
Lower extremity mechanical sketch of the lumbar rehabilitation robot.

As shown in Figure 8 the diagram shows a mechanical sketch of the lower limb of the lumbar rehabilitation robot.

First, calculate the geometric angular relationship of each connecting rod (Formulas 13–16);

$$AD=\frac{BD}{\sin\left(\theta_{0}+\theta_{1}\right)}$$ (13)

$$AB=\frac{BD}{\tan\left(\theta_{0}+\theta_{1}\right)}$$ (14)

$$DE=L-AB;CD=l_{1}-AD$$ (15)

$$\cos\theta =\frac{DE^{2}+l_{2}^{2}-CD^{2}}{2DE\cdot l_{2}}$$ (16)

Where E is the fixed end of the leg support plate; C is the connecting hinge point between the connecting rod and the support plate; A is the connection point between the piston end of the electric push rod and the connecting rod; D is the intersection of the connecting rod straight line and the horizontal line passing through E; B is the intersection of the horizontal line of point a and the vertical line of point D; $\theta_{0}$ is the installation angle between the electric push rod and the base; $\theta_{1}$ is the included angle between the push rod and the connecting rod. $\theta_{2}$ is the relative horizontal attitude angle of the support plate.

## 3 Man machine coupling analysis based on opensim

In this chapter, OpenSim Christophy et al. (2012), Fang and Yuan (2020), a human kinematics simulation software featuring detailed bone and muscle models, was utilized. This software assesses alterations in joint and muscle parameters during lumbar rehabilitation robot usage through simulation. It aims to validate the robot’s efficacy in aiding patients with lumbar rehabilitation training. The chapter’s objective is to create a coupling model between the human body and the lumbar rehabilitation robot using this software, confirming the device’s feasibility. This will offer insights for future development and enhancements.

### 3.1 Coupling model construction of lumbar rehabilitation robot

**FIGURE 9:**
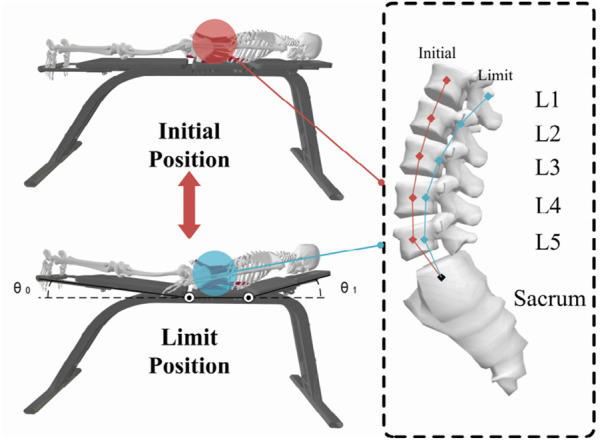
Human-robot coupling model.

To validate the lumbar rehabilitation robot’s efficacy in assisting patients, establishing a coupled human-robot model in the OpenSim environment is crucial (refer to Figure 9). Initially, the robot model was converted to the OpenSim-compatible TSL format via Solidworks, and each mechanical component was integrated into the OpenSim human body model. Subsequently, the human body must be connected to the device using an appropriate method. Initially, the human body’s position within the device is fixed, and a WeldJoint is created at the center of the human abdomen and the abdominal support plate to restrict relative displacement and enable positioning. Additionally, to synchronize the movements of the human legs and chest with the device, a Point On Line Constraint is applied at the interaction points, ensuring alignment with the support plate’s upper plane. This setup simulates the actual movement of the human body during device usage.

As shown in Figure 9, the abdomen and support plate are positioned with weldjoint; The chest/leg contact is constrained by point on line; The contact is a linear spring damping model.

**FIGURE 10:**
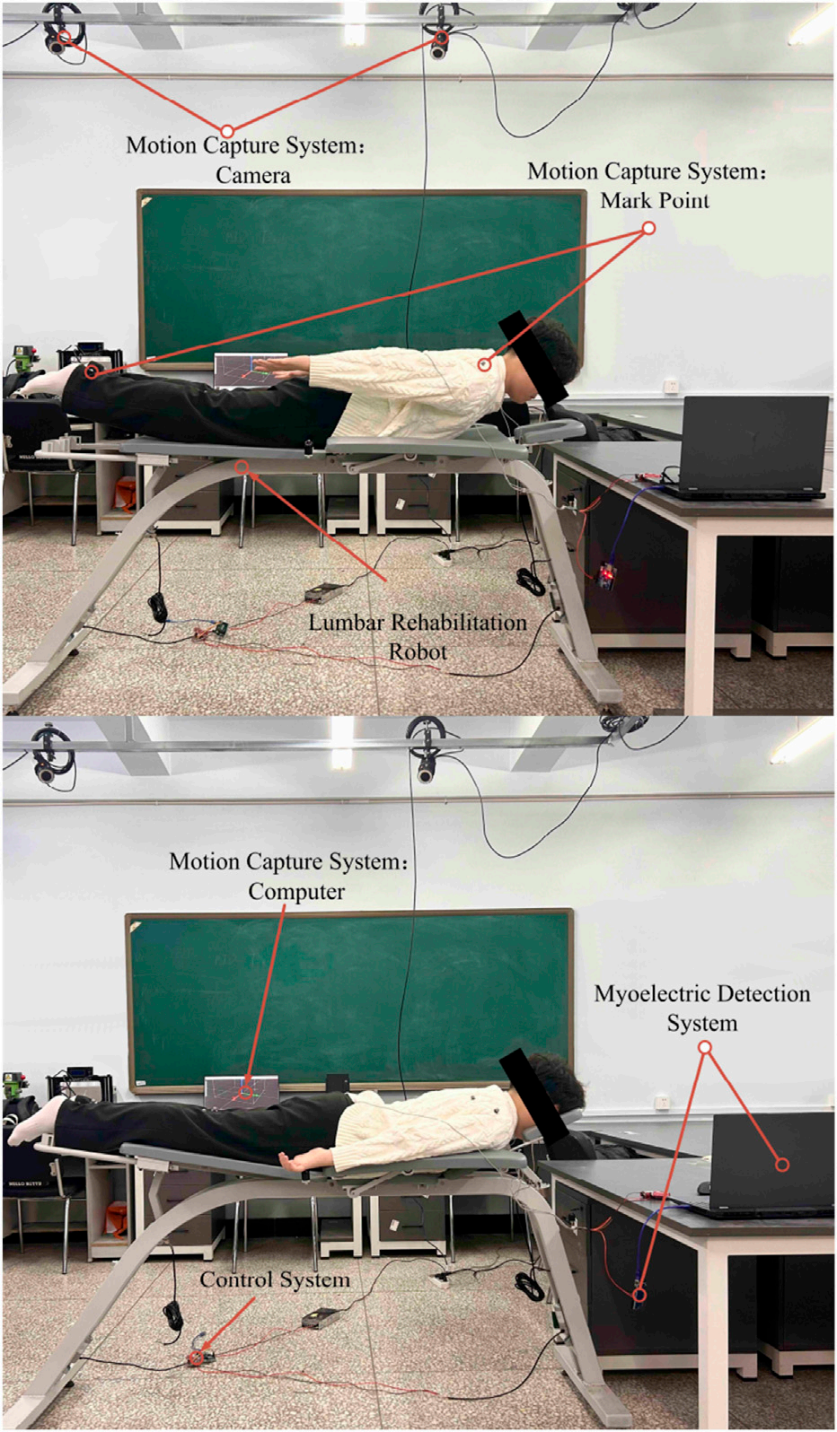
Motion capture.

After creating the human-computer coupling model, the motion trajectories from the motion capture experiment (e.g., Figure 10) were imported as a source file. OpenSim’s Scale Model and Inverse Kinematics tools were then used to align the musculoskeletal model with the experimenter’s body type and reproduce the rehabilitation training maneuvers executed during the experiment.

To examine the lumbar muscle training facilitated by the lumbar rehabilitation robot, we employed two motion capture methods. Firstly, the human body underwent autonomous lumbar rehabilitation training. Secondly, the two electric actuators of the robot applied a force of 200N to the human body individually. Motion data was captured using these methods to establish a basis for studying the activity patterns of the human lumbar muscles.

In order to consider the differences in height, weight and lumbar flexibility between subjects, the Rajagopal 2016 whole body muscle bone model was used in opensim, and scale–IK was performed according to the subject calibration data. Trunk pelvic joints retain the main DOF of flexion and extension, lateral flexion and axial rotation locking/limiting; Pelvis - fixed on the ground, with chest/leg support connected to the human body through point on line constraints; Linear spring damping model for human-machine contact. The robot applies an equivalent auxiliary force on the chest and leg, and the direction is along the normal of the support plate, using a smooth slope time history. The motion capture sampling rate is 120 Hz, and it passes through the fourth-order zero phase Butterworth low-pass; IK weighting and RMS threshold are the same as above. Muscle output was obtained by muscle analysis; The angle is expressed in Rom and the external force is normalized in N/kg. The integrator and tolerance are the same as above.

### 3.2 Study on muscle activity characteristics of coupled model of lumbar

Developing lumbar spine stability is a key aspect of rehabilitation. It involves the lumbar spine’s ability to maintain its structural integrity under load, comprising five vertebrae, adjacent small joints, intervertebral discs, and ligaments to create a motion segment.

Analyzing the lumbar spine stabilization system is essential for designing stabilityfocused rehabilitation programs. This system includes passive, active, and neural subsystems Radziminska et al. (2017) Studnicka and Ampat (2023), with the active subsystem - comprising muscles and tendons - being the focal point of this discussion. The core muscle groups responsible for lumbar spine movement and stability, such as the Rectus abdominis, Multifidi, Internal Oblique Abs, and Quadratus Lumborum Araujo et al. (2022), play a crucial role. To investigate the mechanical structure’s supportive role in lumbar rehabilitation training, As shown in Table 2. This chapter will utilize a human-robot coupling model to gather joint and muscle data for comparison with the patient’s physiological state.

**TABLE 2:** Target muscles Table in lumbar rehabilitation programs.

Flesh	Rectus abdominis muscle	Multifidus muscle	Internal oblique muscle	Lumbar muscle
Placement	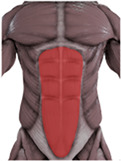	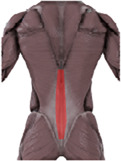	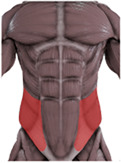	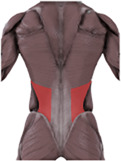
Major Function	Trunk flexion pelvic retroversion participation in abdominal pressure regulation	Intersegmental extensor anti rotation anti shear	Ipsilateral rotation lateral flexion of trunk combined with abdominal pressure	Ipsilateral flexion of trunk pull-down of thorax

### 3.3 Coupled model joint characterization study

**FIGURE 11:**
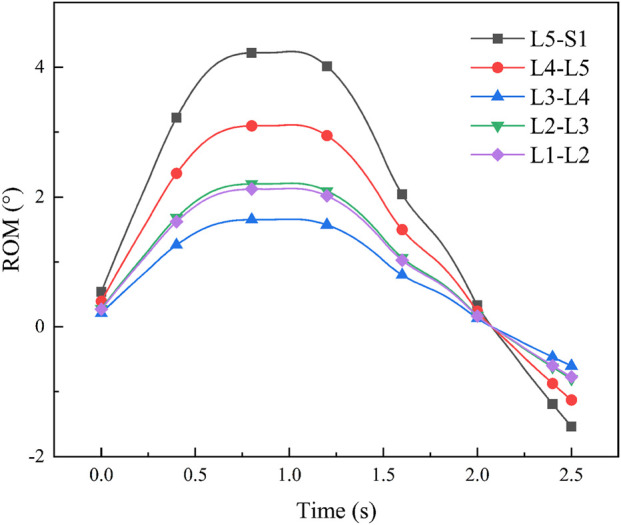
Man-machine coupling model lumbar spine joint angle.

To validate the human-machine coupling model’s accuracy, it is essential to confirmif the lumbar joint angle changes in the model align with real-world movements. This involves utilizing Opensim’s human kinematics tool to capture motion data, enabling the model to mimic the test subject’s lumbar rehabilitation exercises. Subsequently, we will extract and analyze the lumbar spine joint angles from the model in depth (refer to Figure 11 for specifics).

**FIGURE 12:**
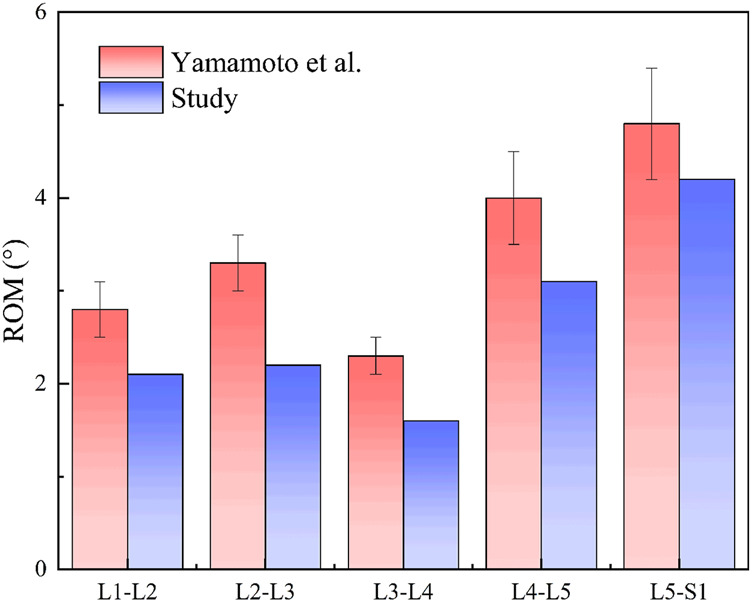
Comparison of lumbar spine angles.

After determining the joint angles of the human-computer coupled lumbar spine, we conducted a comparison with previous theoretical studies. Yamamoto I et al. examined human lumbar spine posterior extension mobility Yamamoto et al. (1989), applying a 10 N-m force to induce lumbar spine bending. Their study yielded average angle values: L1-L2 at 2.8 ° , L2-L3 at 3.3 ° , L3-L4 at 2.3 ° , L4-L5 at 4.0 ° , and L5-S1 at 4.8 ° . Our study’s lumbar spine angles were then compared to these results (refer to Figure 12 for specifics). The comparison indicated alignment between the joint angles of the human-machine coupling model and Yamamoto I et al.’s findings, providing a basis for subsequent muscle analysis.

Through experiments, the average angle of lumbar joint L1-L2, L2-L3, L3-L4, L4-L5 and L5-S1 of the man-machine coupling model is 2°, 2.1°, 1.8°, 3.1°, and 4.0°, respectively.

The potential impact of small deviations on rehabilitation outcomes. Although the extension angle of the human-machine coupling model is consistent with the classical research (Yamamoto et al., 1989), we still notice that there will be small deviations from several sources in practical application (such as IK residuals introduced by motion capture marker positioning and soft tissue deformation, poor ROM allocation caused by individual differences, and compliance errors of constraint models). These deviations may be magnified step by step through the path of “posture segmentation angle muscle strength prescription parameters”: for example, the slight increase in the angle of L4 – S1 may increase the demand for rectus abdominis and quadratus psoas muscle, thus affecting the setting of FS , Rom and holding time of the prescription. In order to reduce the propagation of deviation, we adopted scale–IK registration, point on line contact constraint, low-pass filtering and IK RMS threshold control in the opensim process, and buffered the local peak fluctuations caused by individual differences through Rom and N/kg normalization in the prescription layer, so as to limit the impact of small deviation on the training load and safety boundary within an acceptable range.

Since the work in the past decade has put more emphasis on traceable assistance and quantifiable burden reduction under the conditions of actual tasks and individual differences. For example, Li et al. Used adaptive predictive control in hybrid attitude lift, and Kim used sEMG to verify the feasibility of “progressive load control.” In general, contemporary progress has focused on task related load following control, EMG/mechanics quantitative closed-loop and individualized prescription. This paper is docking this trend in man-machine coupling modeling and training parameterization.

**FIGURE 13:**
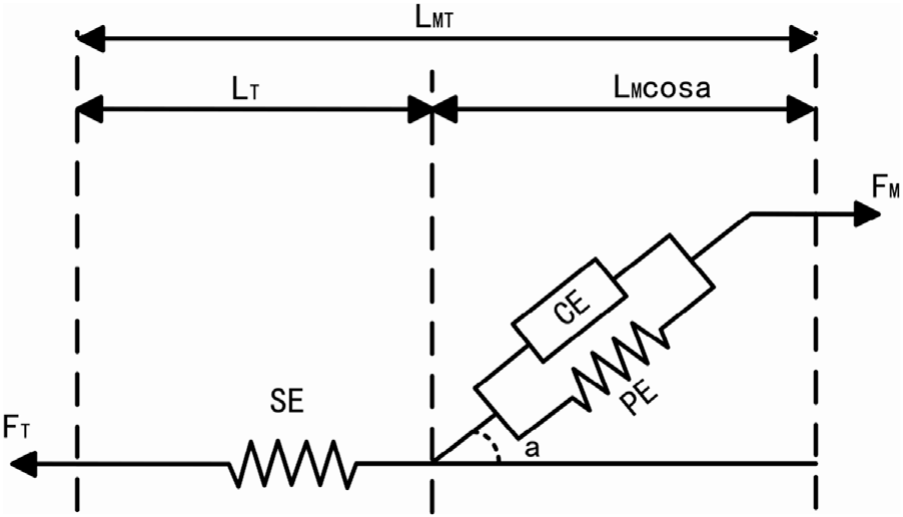
Hill’s three-element model of muscle.

At the core of Opensim’s human muscle analysis theory lies Hill’s three-element model of muscle and Hill’s equation Kojic et al. (1998). Hill introduced a muscle mechanics model in 1938, comprising three elements: Contractile Element (CE), Series Elastic Element (SE), and Parallel Elastic Element (PE), to elucidate muscle force generation. The muscletendon system integrates CE and PE linked to SE through the pinnation angle α , as illustrated in Figure 13, where FM represents muscle tension and FT denotes tendon force. CE actively contracts, driving muscle work, while PE and SE undergo passive contraction and elongation, respectively, during muscle fiber activity. The relationship among muscle-tendon length LMT, tendon length LT, and normalized muscle fiber length LM can be expressed as Formula 17.

$$L_{MT}=L_{T}+L_{M}\cos\alpha \;$$ (17)

**FIGURE 14:**
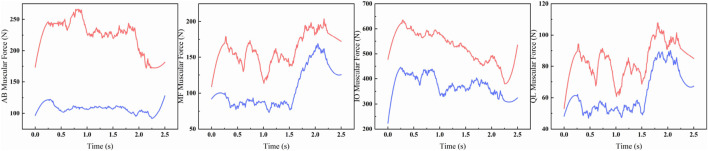
Muscle force comparison curve between musculoskeletal model (red) and human-machine coupled model (blue).

Assessing the efficacy of rehabilitation training in patients using a lumbar rehabilitation robot hinges on monitoring muscle force variations. Human muscle forcegeneration relies on three key factors: activation level, muscle unit length, and muscle unit velocity. This study delves into the physiological changes in patients’ lumbar muscles during device-assisted swallow rehabilitation training. Specifically, it focuses on four crucial muscle groups for lumbar spine stabilization: Rectus Abdominis, Multifidi, Internal Oblique Abs, and Quadratus Lumborum. Among these, the Quadratus Lumborum is the primary focus of analysis. To examine muscle force variations in the human body during autonomous and passive lumbar rehabilitation training with a rehabilitation robot, we utilized the OpenSim muscle analysis tool for data analysis in both modes (refer to Figure 14). Analysis results depicted in Figure 4 reveal that muscle force levels during passive robot-assisted training are notably lower than those during autonomous training, with similar trends in force changes observed in both scenarios. This analysis underscores how the musculoskeletal and human-robot coupling models constructed using OpenSim offer a more intuitive representation of the present operational efficacy and future optimization trajectory for the lumbar rehabilitation robot.

The robot applied 200 N equivalent auxiliary force on the chest/leg respectively; Blue = coupling model (auxiliary), red = musculoskeletal model (autonomous). The quantitative differences of peak variation, Mae and RMSE are shown in Table 3.

**TABLE 3:** Quantitative table of muscle strength curve difference between autonomous training and robot assisted training.

Muscle group	Peak force change (%)	MAE	RMSE
Rectus abdominis	106.6071	124.9910	125.8799
Multifidus muscle	17.5017	56.5538	125.8799
Internal oblique muscle of abdomen	42.6362	152.2354	161.6666
Quadratus lumbalis	19.9394	20.3600	23.2086

Table 3 summarizes the curve differences of the four lumbar stabilizing muscles under the conditions of the autonomous training and the robot assisted, using three indicators of peak force change (amplitude,%), Mae and RMSE. The results showed that the difference between rectus abdominis (peak force change 106.61%, MAE 124.99, RMSE 125.88) and multifidus (17.50%, 52.36, 56.55) was the largest, and the difference between internal oblique abdominis and quadratus lumboris was 42.64% (152.24161.67) and 19.94% (20.36, 23.21), respectively. The overall range is 17.50%–106.61% of the peak force change, MAE 20.36–152.24,RMSE 23.21–161.67.

Based on the above parameterization, the system can cover three types of prescriptions: 1. Passive assisted (high Fs Low Rom , low ω ) for pain/early tolerance training; 2. Active auxiliary (medium Fs , medium/high Rom ) for endurance and coordinated recovery; 3. Resistance/reduction (set the virtual damping/stiffness in the impedance model as the resistance direction and limit the peak force) for late strength and control training. Consistent with the results of “auxiliary reduction of muscle strength demand and consistent trend” shown in this article, adjust Fs , Rom and ω can smoothly transition different protocols within the same security boundary.

## 4 Experiment and results

In this study, we propose a design scheme for a lumbar rehabilitation robot focused on swallow rehabilitation training. We conduct kinematic forward and inverse solution analyses, along with kinetic analysis, to validate the robot’s functionality. Subsequently, we determine the robot’s workspace, setting the stage for human-robot coupling experiments.In addition, the protocol Parameterization Based on Fs – ROM – ω enables the device to continuously transition between passive auxiliary, active auxiliary and resistance/reduction assistance according to the prescription, which is convenient for clinical individualized use.

Aiming at the problem that the current lumbar rehabilitation robot has a long iteration cycle and can not directly analyze its impact on human musculoskeletal, the impact of lumbar rehabilitation robot on human physiology is studied. A musculoskeletal model including lumbar vertebrae was built in opensim human kinematics environment, and the influence of lumbar rehabilitation robot on musculoskeletal model with or without assistance was analyzed from the perspective of muscle strength change. The results show that the lumbar rehabilitation robot not only meets the effect of rehabilitation training, but also takes into account the safety of patients, especially for weak and elderly patients.

Through the simulation study of man-machine coupling model, it provides theoretical support for the optimal design of lumbar rehabilitation robot. Although this study verifies the effectiveness and safety of the designed lumbar rehabilitation robot, there are still some limitations. The load capacity of the electric push rod drive is limited, and its adaptability to patients with extreme weight needs to be verified; The current security protection mechanism mainly depends on force and angle threshold setting, and there is still room for improvement in response to sudden abnormal posture; The real-time response performance of the controller when switching between different motion modes also needs to be further improved. The follow-up research will focus on these problems in the aspects of structural optimization, intelligent safety protection and control algorithm iteration, so as to further improve the clinical applicability. Therefore, future research will not only focus on Mechanism Optimization and control algorithm iteration, but also follow the route of “prototype manufacturing and laboratory validation - preliminary test of healthy subjects - small sample preclinical trial - multi center clinical application promotion”, so as to realize the smooth transition of the system to the actual clinical deployment.

With the flexibility of mechanical support and prescription parametric control, the robot has the potential of cross scene application, and can provide a unified training platform for low back pain and postoperative spinal rehabilitation.

## Data Availability

The original contributions presented in the study are included in the article/supplementary material, further inquiries can be directed to the corresponding author.
